# Patatin-like phospholipase domain–containing protein 3 promotes transfers of essential fatty acids from triglycerides to phospholipids in hepatic lipid droplets

**DOI:** 10.1074/jbc.RA118.002333

**Published:** 2018-03-19

**Authors:** Matthew A. Mitsche, Helen H. Hobbs, Jonathan C. Cohen

**Affiliations:** From the ‡ Departments of Molecular Genetics and Internal Medicine,; § Center of Human Nutrition, and; ¶ Howard Hughes Medical Institute, University of Texas Southwestern Medical Center, Dallas, Texas 75390

**Keywords:** triglyceride, phospholipid, phosphatidylglycerol, polyunsaturated fatty acid (PUFA), lipase, hepatic steatosis

## Abstract

Fatty liver disease (FLD) is a burgeoning health problem. A missense variant (I148M) in patatin-like phospholipase domain–containing protein 3 (PNPLA3) confers susceptibility to FLD, although the mechanism is not known. To glean first insights into the physiological function of PNPLA3, we performed detailed lipidomic profiling of liver lysates and lipid droplets (LDs) from WT and *Pnpla3*^−/−^ (KO) mice and from knock-in (ki) mice expressing either the 148M variant (IM-ki mice) or a variant (S47A) that renders the protein catalytically inactive (SA-ki mice). The four strains differed in composition of very-long-chain polyunsaturated fatty acids (vLCPUFA) in hepatic LDs. In the LDs of IM-ki mice, vLCPUFAs were depleted from triglycerides and enriched in phospholipids. Conversely, vLCPUFAs were enriched in triglycerides and depleted from phospholipids in SA-ki and *Pnpla3*^−/−^ mice. Release of vLCPUFAs from hepatic LDs incubated e*x vivo* was increased in droplets from IM-ki mice and decreased from droplets isolated from *Pnpla3*^−/−^ and SA-ki mice relative to those of WT mice. Thus, the physiological role of PNPLA3 appears to be to remodel triglycerides and phospholipids in LDs, perhaps to accommodate changes in LD size in response to feeding. Because SA-ki and IM-ki both cause FLD and yet have opposite effects on the lipidomic profile of LDs, we conclude that the FLD associated with genetic variation in PNPLA3 is not related to the enzyme's role in remodeling LD lipids.

## Introduction

A missense variant (I148M) in patatin-like domain–containing protein 3 (PNPLA3)^3^ is the strongest genetic risk factor yet identified for nonalcoholic and alcoholic fatty liver disease (FLD) (1, 2). The variant is associated with an increase in hepatic triglyceride (TG) content (HTGC) (1) as well as steatohepatitis (3, 4), cirrhosis (5, 6), and hepatocellular carcinoma (5, 7, 8). The effect of PNPLA3(148M) on FLD is greatly amplified by obesity and insulin resistance (9–11). Despite this strong, reproducible association, the pathogenic mechanism underlying the relationship between PNPLA3(148M) and FLD has not been fully elucidated.

PNPLA3 is expressed at the highest levels in liver and adipose tissue and is strongly up-regulated by feeding and reduced by fasting (12–14). Within hepatocytes, >90% of PNPLA3 resides on lipid droplets (LDs) (15). Purified recombinant PNPLA3 has TG hydrolase activity (16, 17), but not phospholipase or retinyl esterase activity (16–18). The I148M substitution reduces *in vitro* TG hydrolytic activity by ∼80% (18). These findings led to the hypothesis that the I148M substitution inactivates PNPLA3, thus reducing lipolysis of TG in hepatocytes and causing hepatic steatosis (18).

This hypothesis proved to be incompatible with the subsequent finding that genetic deficiency of PNPLA3 in mice fails to result in hepatic steatosis (19, 20). Therefore, PNPLA3(148M) is not associated with increased HTGC due to a simple loss of function. To further assess the mechanism by which PNPLA3(148M) promotes FLD, we generated transgenic mice expressing human PNPLA3(WT) or PNPLA3(148M) in liver or in adipose tissue (21). No changes in body fat content or hepatic TG levels were seen when the transgenes were expressed in adipose tissue. Hepatic overexpression of PNPLA3(WT) also had no effect on liver TG levels. Thus, the mutation is not a pure gain-of-function mutation. In contrast, hepatic overexpression of PNPLA3(148M) increased liver TG levels 2–3-fold in chow-fed mice (21). These data are consistent with PNPLA3(148M) being a neomorph that confers a new function, resulting in hepatic steatosis.

To mitigate potential artifacts caused by overexpressing human PNPLA3 in mice, we introduced the I148M substitution into the endogenous mouse *Pnpla3* gene (22). On a chow diet, hepatic TG levels were comparable in WT and IM-ki mice (22). Feeding a fat-free, high-sucrose diet (HSD) increased hepatic TG in both strains of mice relative to chow diet, but the increase was significantly greater (2–3-fold) in the IM-ki animals. Similar results were obtained in mice expressing the catalytically dead enzyme (SA-ki mice) (22). No evidence was found to support the notion that PNPLA3(148M) or PNPLA3(47A) induces endogenous synthesis of TG in the liver (23). Rather, accumulation of PNPLA3(148M) on hepatic LDs appears to interfere with TG mobilization (21, 23).

Whereas hepatic overexpression of PNPLA3(WT) does not cause HTGC (21), it does change the distribution of fatty acids (FAs) in the liver. Hepatic overexpression of PNPLA3(WT) or PNPLA3(148M) is associated with depletion of very-long-chain polyunsaturated FAs (vLCPUFAs) in hepatic TG (21). A similar depletion of vLCPUFA in TG was seen in cultured hepatocytes expressing recombinant PNPLA3(WT) or PNPLA3(148M) (24). To determine how the alteration in hepatic lipid composition is related to hepatic steatosis, we analyzed the composition and flux of hepatic FAs in WT, *Pnpla3*^−/−^ (KO), SA-ki, and IM-ki mice fed a HSD to promote *de novo* lipogenesis. Here, we show that inactivation of PNPLA3 by genetic ablation or by substitution of the catalytic serine with alanine (S47A) resulted in accumulation of vLCPUFA in TG and depletion of vLCPUFA from the PL of LD. In contrast, the partitioning of vLCPUFA from TG to PL was increased in hepatic LD from the 148M-ki animals. Analysis of FA release from hepatic LDs that were incubated *ex vivo* showed similar rates of palmitoleate and oleate release in the four strains, whereas the release of arachidonate and docosahexaenoic acid was decreased in LDs from KO and SA-ki animals and increased in LDs from IM-ki mice. These findings indicate that PNPLA3 catalyzes the transfer of vLCPUFA from TG to PL in LD. This activity serves to remodel the lipids in hepatic LD but does not explain the hepatic steatosis associated with PNPLA3(148M).

## Results

### FA composition of total hepatic lipids did not differ among WT, IM-ki, SA-ki, and KO mice

**Figure 1.**
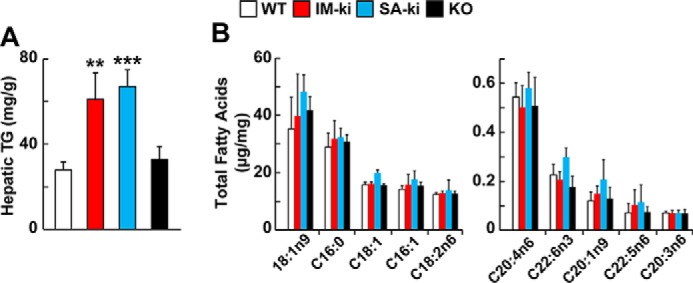
**Hepatic lipid composition of genetically modified PNPLA3 mice.** *A*, liver TG content of WT (*white*), PNPLA3 I148M knock-in (*IM-ki*; *red*), PNPLA3 S47A knock-in (*SA-ki*; *blue*), and PNPLA3 knockout (*KO*; *black*) male mice (*n* = 4–6/group, 12 weeks old). In this and all other experiments, mice were entrained for 3 days by fasting from 6 p.m. to 8 a.m. and fed a HSD from 8 a.m. to 6 p.m. and killed 4 h into the last refeeding cycle. TGs were measured using enzymatic assays. *B*, total hepatic FA composition of genetically modified male mice fed a HSD for 4 weeks (*n* = 4–6/group, 12 weeks old). Liver lipids were hydrolyzed and derivatized with trimethylsilane and then measured by GC-flame ionization detection. The data are expressed per mg of liver. The experiment was repeated twice, and the results were similar. *Error bars*, S.D. **, *p* < 0.01; ***, *p* < 0.001.

TG levels were measured in lipid extracts of liver homogenates from WT, IM-ki, SA-ki, and KO mice fed a HSD for 4 weeks. As reported previously, hepatic TG levels were 2-fold higher in the IM-ki and SA-ki mice than in their WT littermates (22) (Fig. 1*A*). The HTGCs of KO and WT animals were similar, which is consistent with prior reports (19, 20). The FA composition of whole-lipid extracts did not differ significantly among the strains (Fig. 1*B*). Thus, the expression of PNPLA3 does not appear to have a major effect on uptake, elongation, or desaturation of FAs by the liver.

### Enrichment of vLCPUFAs in hepatic TG of KO and SA-ki mice

**Figure 2.**
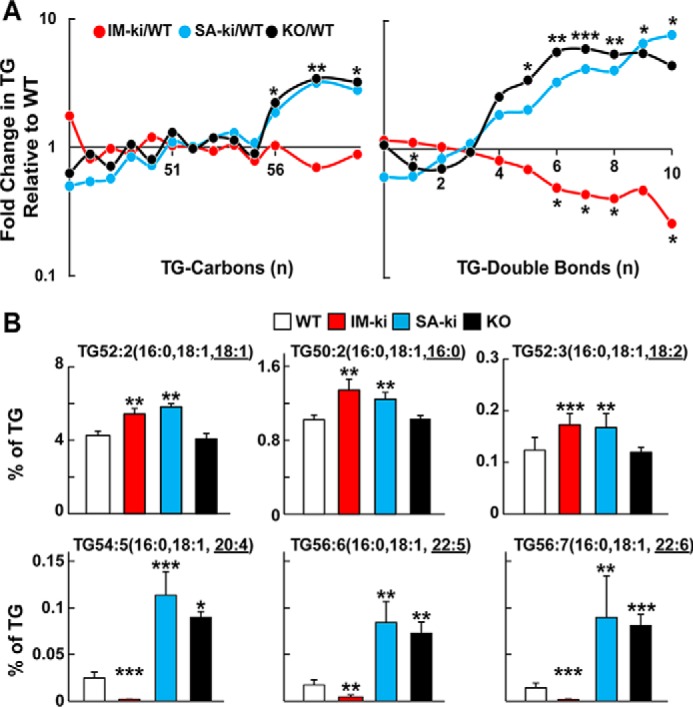
**Hepatic TG composition in genetically modified PNPLA3 mice.** *A*, the number of carbons (*left*) and number of double bonds (*right*) in hepatic TGs of IM-ki (*red*), SA-ki (*blue*), and KO (*black*) mice relative to their WT counterparts. Male mice were fed a HSD for 4 weeks (*n* = 6–8/group, 14 weeks old) and then sacrificed after 4 h of refeeding. Hepatic TGs were analyzed by direct infusion lipidomics. TGs were grouped based on number of FA carbons (*left*) or double bonds (*right*), as determined by the MS/MS signal from untargeted lipidomics analysis. Values were normalized to the total lipid signal and expressed relative to the corresponding value in WT mice. The data are plotted on a log-normal scale. *B*, selected TGs were measured by LC-MS/MS analysis. Shown are representative examples of abundant TGs (*top*) and vLCPUFA-containing TGs (*bottom*) in livers of PNPLA3 genetically modified mice. Mass pairs for identification of abundant TG were 876.7 and 577.6, 848.7 and 577.6, and 874.7 and 577.6 Da for the TGs shown *left* to *right*, respectively, and 898.8 and 577.7, 924.8 and 577.7, and 922.8 and 577.7 for the vLCPUFA-containing TGs shown *left* to *right*, respectively. Underlined FAs indicate the neutral loss of the measured species. Values were normalized to the sum of all measured TG signals. Liver TG analysis was repeated twice with similar results. *Error bars*, S.D. *, *p* < 0.05; **, *p* < 0.01; ***, *p* < 0.001.

To determine the effect of PNPLA3 on the FA composition of hepatic glycerolipids and glycerophospholipids, we measured the FA content of these lipids using untargeted direct infusion MS/MS^ALL^ lipidomics. A total of 700–800 lipids and ∼12,000 lipid features were identified (Table 1). The most significant and consistent differences in FA composition among the mouse strains were in TGs (Fig. 2). To illustrate these differences, the relative -fold changes in TG-FAs between the IM-ki, SA-ki, and KO mice and the WT animals were plotted based on the number of TG-carbons (Fig. 2*A*, *left*) or the number of double bonds (Fig. 2*A*, *right*) in each TG species. Chain lengths of the TG-FAs in IM-ki mice were similar to those of the WT animals, whereas TG-FAs with a combined chain length of ≥56 carbons were enriched in the SA-ki and KO mice (Fig. 2*A*, *left*). The IM-ki mice were depleted of TG containing vLCPUFAs when compared with the WT animals, whereas the SA-ki and KO mice were enriched in hepatic TGs containing FAs with ≥5 double bonds (Fig. 2*A*, *right*). The reduction in TG-vLCPUFAs in the IM-ki mice was similar to what was observed previously in mice expressing a PNPLA3(148M) transgene and in cells overexpressing PNPLA3(148M) (21).

**Table 1:** **Mass features identified from untargeted lipidomics of WT mouse liver**

Lipid class	Percentage of lipids	Number observed
Triglycerides	17.89	433
Diacylglycerides	0.46	84
Cholesteryl esters	0.53	12
Monoacylglycerides	0.04	4
Fatty acid esters of hydroxyl fatty acids	0.03	14
Phosphatidylcholines	48.8	103
Phosphatidylethanolamines	2.7	79
Phosphatidylserines	4.1	39
Phosphatidylglycerols	1.7	26
Phosphatidylinositols	0.35	14
Lyso-phosphatidylcholines	0.4	17
Lyso-phosphatidylethanolamines	0.08	4
Sphingomyelins	0.73	18
Free fatty acids	0.44	15

We confirmed these differences in TG-FA profiles using targeted LC-MS/MS. The most abundant TGs in the mouse livers were TG52:2 (16:0, 18:1, 18:1), TG 50:2 (16:0, 18:1, 16:0) and TG52:3 (16:0, 18:1, 18:2) (Fig. 2*B*). (Underlined FAs indicate the neutral loss of the measured species.) TGs containing these FAs were significantly more abundant in the IM-ki and SA-ki mice, reflecting the increase in total TG in the livers of these animals (Fig. 2*B*, top). Major differences in TG-FA abundance were seen in the subset of TG that contained at least one vLCPUFA (Fig. 2*B*, bottom). Levels of vLCPUFA-containing TGs were significantly higher in the livers of SA-ki and KO mice than in either the WT or IM-ki animals. For example, TG-FAs with 16:0, 18:1, and either arachidonic acid (20:4) (Fig. 2*B*, *bottom left*), docosapentaenoic acid (DPA; 22:5) (Fig. 2*B*, *bottom middle*), or docosahexanoic acid (DHA; 22:6) (Fig. 2*B*, *bottom right*) were significantly more abundant in SA-ki and KO mice than in the WT or IM-ki mice. The IM-ki mice had significantly fewer vLCPUFA-containing hepatic TGs relative to total lipids than did the WT mice.

Thus, although both SA-ki and IM-ki sucrose-fed mice have increased HTGC (22), the pattern of changes in TG-FA composition differed between the two strains. The enrichment pattern of TG- vLCPUFAs in the SA-ki mice was similar to that of the KO mice, not the IM-ki mice (Fig. 2). PNPLA3 appears to play a role in the partitioning of vLCPUFAs among lipids in hepatic LDs, but disruption in this pathway appears not to be causally related to the increase in HTGC associated with PNPLA3(148M).

### FA composition of cholesteryl esters (CEs), retinyl ester (REs), and major classes of phospholipids (PLs) in liver lysates of SA-ki, IM-ki, KO, and WT mice

**Figure 3.**
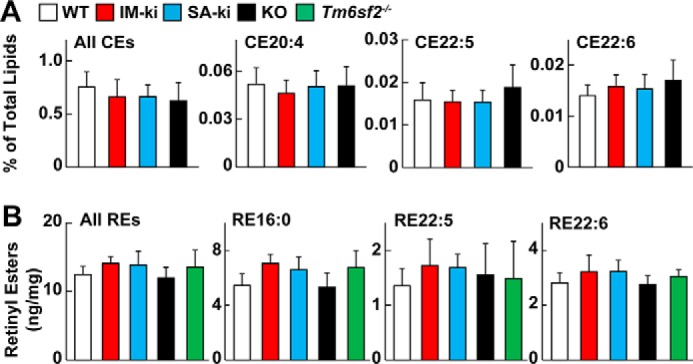
**FA composition of hepatic CEs and REs.** *A*, levels of hepatic CEs (all CEs) and most abundant polyunsaturated CEs in PNPLA3(WT) (*white*), IM-ki (*red*), SA-ki (*blue*), and KO mice (*black*). Male mice (*n* = 6–8/group, 14 weeks old) were fed a HSD for 4 weeks and killed 4 h after refeeding. CEs were analyzed by direct infusion lipidomics and normalized to total lipid signal. Liver CE analysis was repeated twice with similar results. *B*, total REs (all REs) and the most abundant REs were measured in male mice fed a HSD for 4 weeks (*n* = 4–6/group, 12 weeks old). REs were measured using LC-MS/MS with RE17:0 as an internal standard. Total REs were determined by summing the intensity of all mass features containing an MS2 fragment of 269.2 Da with an MS1 mass corresponding to an RE. Values were normalized to the internal standard of RE17:0. The experiment was repeated once, and the results were similar. *Error bars*, S.D.

No significant differences were apparent among the four strains of mice in the FA composition of CEs, the other major hepatic neutral lipid (Fig. 3*A*). Previously, PNPLA3 was implicated as a retinyl esterase; expression of PNPLA3(148M) in cultured cells was associated with an increase in REs (25). We found small, but not statistically significant (*p* < 0.1), increases in REs in both the IM-ki and SA-ki mice relative to WT animals (Fig. 3*B*). We also measured REs in another strain of mice that has fatty liver due to deficiency of TM6SF2, a protein required for lipidation of very low density lipoproteins (26, 27). The increase in liver TG in *Tm6sf2*^−/−^ mice is similar in magnitude to that seen in sucrose-fed IM-ki and SA-ki mice, and the levels of RE in the *Tm6sf2*^−/−^ mice were also similar (Fig. 3*B*). These results raise the possibility that the modest increase in REs in the two KI strains is a consequence of the increase in HTGC rather than a specific effect of the sequence variations on PNPLA3 enzymatic activity.

**Figure 4.**
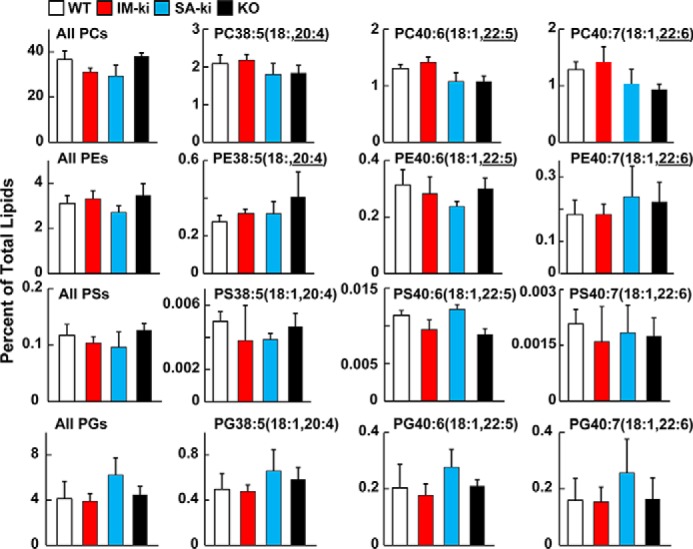
**PL composition of liver lysates.** Male mice (*n* = 6–8/group, 14 weeks old) were fed a HSD for 4 weeks and killed after 4 h of refeeding. Total hepatic PL composition was determined by direct infusion lipidomics in WT (*white*), IM-ki (*red*), SA-ki (*blue*), and PNPLA3 KO mice (*black*). PLs were identified by their negative ion. The reported vLCPUFA-containing PLs were identified based on the MS2 value. Similar results were observed in positive mode. All values were normalized to the sum of the positive and negative signal from direct infusion lipidomics. The experiment was repeated twice with similar results. *Error bars*, S.D.

Next we used direct infusion untargeted lipidomics to compare the FA composition of the four major classes of hepatic PLs in liver homogenates by direct infusion untargeted lipidomics: phosphatidylcholine, (PC), phosphatidylethanolamine (PE), phosphatidylserine (PS), and phosphatidylglycerol (PG). No differences in FA composition were observed in the most plentiful species of these four PL classes (Fig. 4). Thus, the increased levels of vLCPUFAs in hepatic TGs from liver lysates of SA-ki and KO mice were not compensated by a decrease in vLCPUFAs in the other two major lipid classes, CEs and PLs (Figs. 2–4), as might have been expected, given the similarities in total vLCPUFA content among the four strains (Fig. 1).

### Untargeted lipidomics reveals that SA-ki and KO LDs have similar lipid profiles

**Figure 5.**
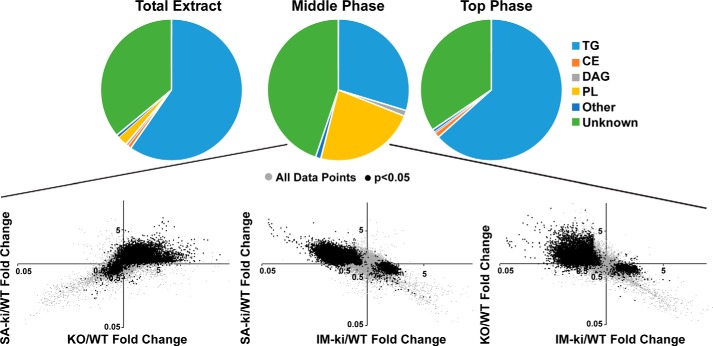
**Lipidomic comparisons of lipid droplets.** *Top*, lipid composition of LDs from a Bligh–Dyer extract compared with the middle and top phase of a three-phase extraction based on direct infusion lipidomics analysis. Lipids were identified based on their tandem mass pairs. Lipid features that were not identified were classified as unknown lipids. Reported values are means of livers (*n* = 4). -Fold change of KO mice relative to WT mice LDs were plotted against the -fold change in SA-ki to WT mice. Hepatic LDs were prepared from male mice on a HSD for 4 weeks (*n* = 5–7/group, 14 weeks old) after 4 h of refeeding. Each point represents the -fold change in KO mice on the *x* axis and SA-KI mice on the *y* axis. Values were normalized to the sum of all lipid signals. The relationship between IM-ki and SA-ki or KO mice relative to WT is also shown. *Black points* had a *p* value < 0.05 in the IM-ki mice. The analysis was repeated once with similar results.

Because PNPLA3 is located predominantly on LDs (23), we performed lipidomics on LDs isolated from the four strains of mice. The vast majority of lipid in LDs is TG, which makes it challenging to measure the content of less abundant lipids, such as PLs. To circumvent this problem, we used a three-phase extraction in which PLs partition predominantly to the middle phase (Fig. 5, *top*)(28).

Using untargeted MS/MS^All^ lipidomics, we measured all MS/MS features in extracts from the middle phase of LDs, which contains most of the PLs, but also a fraction of the TGs as well as other unidentified lipids. For each feature, the -fold change in intensity relative to WT mice was compared. The relative -fold changes in lipids from the LDs of SA-ki mice were positively correlated with those seen in the KO animals (Fig. 5, *bottom left graph*). Thus, as was observed for TG, the two obligate loss-of-function mutations in PNPLA3 had similar effects on hepatic LD composition in mice. In contrast, the -fold change in intensity of lipids from the IM-ki animals varied inversely with those seen in the SA-ki and KO mice (Fig. 5*B*, *bottom middle* and *right graphs*). Thus, the 148M variant appears to confer a gain, rather than a loss, of PNPLA3 activity.

### Genetic variation in PNPLA3 alters PL-FA distribution in LDs

**Figure 6.**
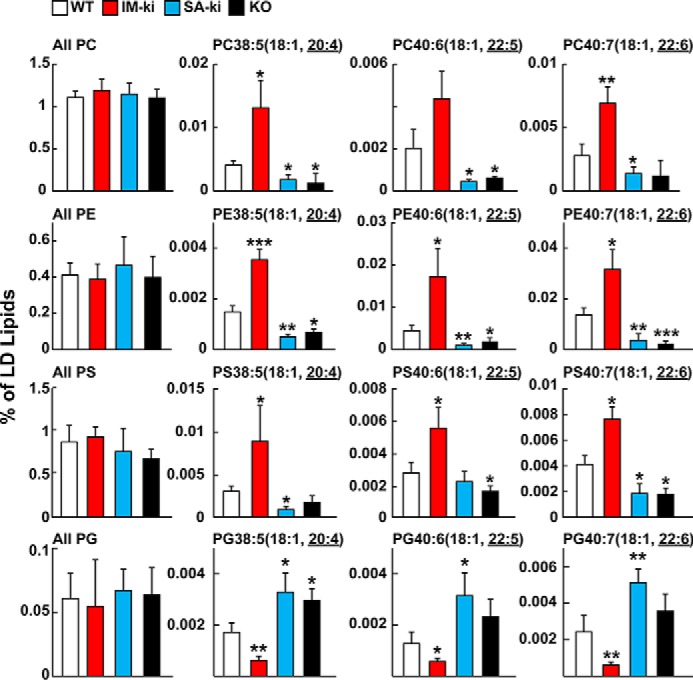
**Composition of PLs of hepatic LDs by untargeted lipidomics.** Male mice (*n* = 5–7/group, 14 weeks old) of the indicated genotype were fed a HSD for 4 weeks and refed for 4 h before tissues were collected. Four PL classes (PCs, PEs, PSs, and PGs) were analyzed by direct infusion lipidomics (see “Experimental procedures”). The total content of each PL class was calculated by summing all unambiguous signals obtained in negative mode. Individual PLs were identified by their negative ion with the MS2 of the vLCPUFA. Similar results were observed in positive mode. All values were normalized to the sum of the positive and negative mode signal, where the majority of the signal was TGs. Analysis was repeated once with similar results. *Error bars*, S.D. *, *p* < 0.05; **, *p* < 0.01; ***, *p* < 0.001.

Total PC, PE, PS, and PG content of the LDs (expressed as a percentage of total LDs) did not differ significantly among the strains (Fig. 6, *left column*). However, major differences were apparent in the vLCPUFA content of PC, PE, and PS between the strains; the relative amounts of these lipids were reduced in LD from SA-ki and KO mice and increased in LD from IM-ki mice (Fig. 6, *right three graphs*).

The differences in FA content of PGs in hepatic LDs from the four strains mirrored those seen in TGs (Fig. 6, *bottom*) in a direction that was similar to that seen in TGs (Fig. 2). For this analysis, the PGs were identified by a neutral loss of 171.1 Da in positive mode or by FA fragment ions in the negative mode. The vLCPUFA content of PGs was decreased in hepatic LDs from IM-ki mice and increased in LDs from SA-ki and KO mice (Fig. 6, *bottom*).

**Figure 7.**
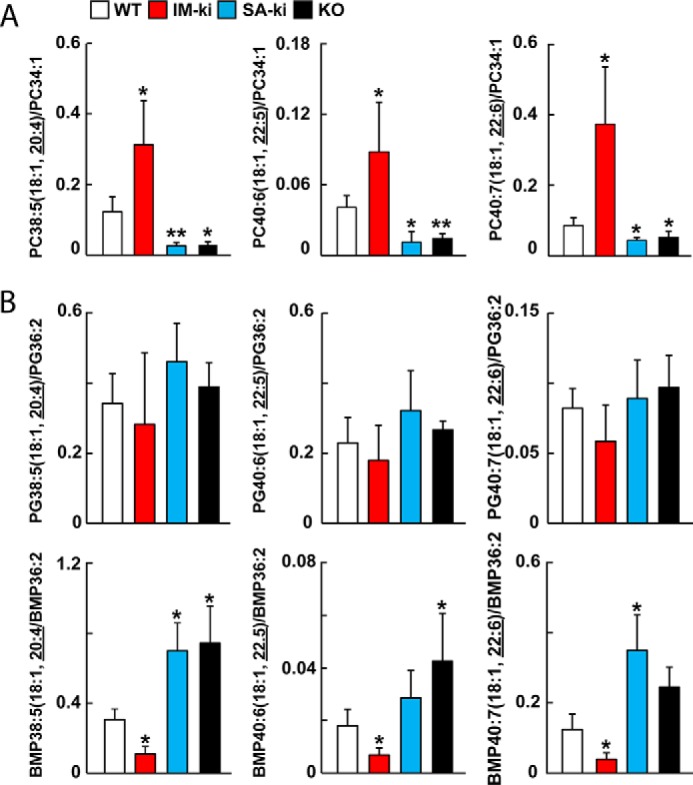
**PL composition of hepatic LDs by targeted LC-MS/MS.** PL composition of hepatic LDs from WT (*white*), IM-ki (*red*), SA-ki (*blue*), and PNPLA3 KO mice (*black*) was analyzed by targeted LC-MS/MS. Male mice (*n* = 5–7/group, 14 weeks old) were fed a HSD for 4 weeks and refed for 4 h before collecting tissues. PCs (*A*), PGs, and BMPs (*B*) were targeted for analysis using LC-MS/MS. Individual PGs were identified by their negative ion with the MS2 of the vLCPUFA. Values were normalized to an abundant PL in the same class, as indicated by the denominator on the *y* axis. Analysis was repeated once, and the results were similar. *Error bars*, S.D.; *, *p* < 0.05; **, *p* < 0.01.

To confirm these findings, the FA compositions of PC and PG were measured using an orthogonal method, targeted LC-MS/MS analysis (Fig. 7). For this analysis, measurements of selected PCs containing vLCPUFA were normalized to the most plentiful endogenous PC (PC34:1(16:0,18:1)) (Fig. 7*A*). The pattern of FA enrichment and depletion in all of the PCs containing vLCPUFA was similar to that seen using untargeted methods (Fig. 6).

We also measured PGs using direct infusion lipidomics, but the results were ambiguous because the mass values were isobaric with bis(monoacylglycerol) phosphates (BMPs). BMPs and PGs have the same elemental composition. To resolve this ambiguity, we used normal-phase LC-MS/MS to separate PGs from BMPs chromatographically before the MS/MS analysis. The relative levels of vLCPUFA-containing PGs normalized to PG36:2(18:1,18:1) in the LDs did not differ significantly among the four strains (Fig. 7*B*, *top*). The relative levels of vLCPUFA-containing BMPs using targeted analysis mirrored the pattern observed using untargeted lipidomics and initially ascribed to PGs (Fig. 7, *bottom*). BMPs containing 20:4, 22:5, and 22:6 FAs were decreased in IM-ki mice and increased in SA-ki and KO mice relative to WT animals. Thus, the pattern and direction of changes in vLCPUFA in BMPs were similar to those seen for TGs (Fig. 2*B*).

Taken together, the changes in FA composition of hepatic lipids are consistent with the notion that PNPLA3 promotes the transfer of vLCPUFAs between TG and PLs in LDs.

### FA sorting in HuH7 cells overexpressing PNPLA3

**Figure 8.**
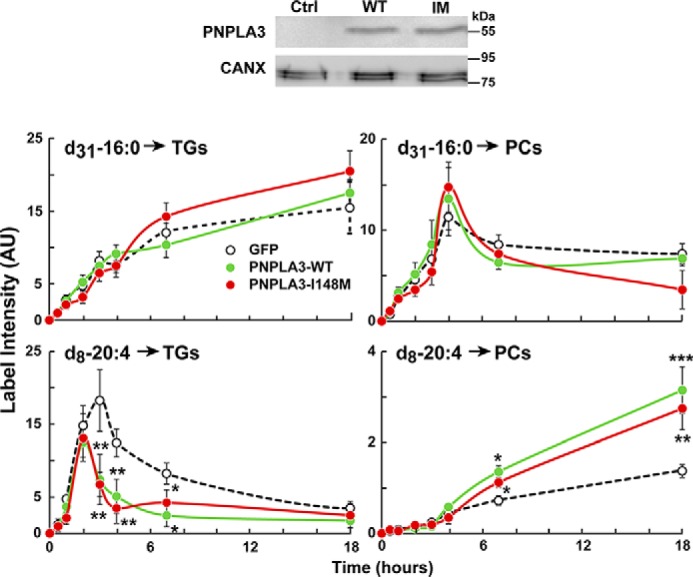
**Synthesis of TG and PL in in HuH7 cells overexpressing PNPLA3.** HuH7 cells were infected with a GFP adenoviruses (GFP, *dotted line*), PNPLA3(WT) (*green*), or PNPLA3(148M) (*red*). After 2 days, palmitate-*d*_31_ and arachidonate-*d*_8_ were conjugated with BSA and added to the medium at a final concentration of 1 μm each. Cells were then harvested at the indicated time points (*n* = 3 dishes/time point). Expression of PNPLA3 constructs was analyzed by immunoblotting using a V5 antibody. Lipids were extracted, and the incorporation of the isotope-labeled FAs into TGs and PCs was measured by direct infusion lipidomics. PCs were identified by the 184-Da MS2 fragment in positive mode. TGs were identified based on their FA neutral loss. Values were normalized to the PC and TG standards in the SPLASH standard mixture. The experiment was repeated twice with similar results. *Error bars*, S.D. *, *p* < 0.05; **, *p* < 0.01; ***, *p* < 0.001.

Intracellular FA sorting is a multienzyme process that leads to enrichment of saturated FAs and monounsaturated fatty acids (MUFAs) in TG and enrichment of vLCPUFAs in PLs (29). To directly assess the effect of PNPLA3 expression on FA sorting, we used stable isotope labeling and MS to measure the rate of incorporation of palmitate (16:0) and arachidonate (20:4) into TGs and PCs in HuH7 cells overexpressing GFP, PNPLA3(WT), or PNPLA3(148M) (Fig. 8, *top*). The rates of incorporation of labeled palmitate-*d*_31_ (16:0) into TGs were linear for the first 5–6 h (Fig. 8, *left*) and continued to increase over the ensuing 12 h. Rates of incorporation were similar in the three groups of cells. The incorporation of palmitate-*d*_31_ into PCs was more rapid and peaked at 5 h (Fig. 8, *right*).

The time course of incorporation of arachidonate-*d*_8_ into TG and PC differed from that seen for palmitate (Fig. 8, *bottom*). The pattern of incorporation of arachidonate into TG resembled the pattern observed for palmitate into PC. In cells expressing PNPLA3(WT), PNPLA3(148M), or GFP, the incorporation of arachidonate-*d*_8_ into TG increased rapidly and reached a maximum at ∼3 h. Thereafter, arachidonate incorporation into TG decreased progressively. The incorporation of arachidonic acid into PC was similar in the three groups of cells over the first 4 h. Subsequently, the IM-ki cells incorporated significantly more labeled arachidonic acid into PC (Fig. 8, *bottom right*). At the end of the experiment, the incorporation of label into PCs was 2–3-fold higher in the IM-ki cells than in the cells expressing GFP.

The data shown in Fig. 8 support a model in which PNPLA3 promotes a reduction in the vLCPUFA content of TGs (Fig. 2) and an increase in the vLCPUFA content of PC (Fig. 6). The data are consistent with the notion that PNPLA3, an enzyme with TG hydrolase activity (18), removes vLCPUFAs from TGs and transfers them to lyso-PC or delivers them to lysophospholipid acyltransferase to form PC. To test this hypothesis, we next examined the dynamics of lipid remodeling in LDs isolated from the liver of the genetically modified mice.

### LD FA release ex vivo

**Figure 9.**
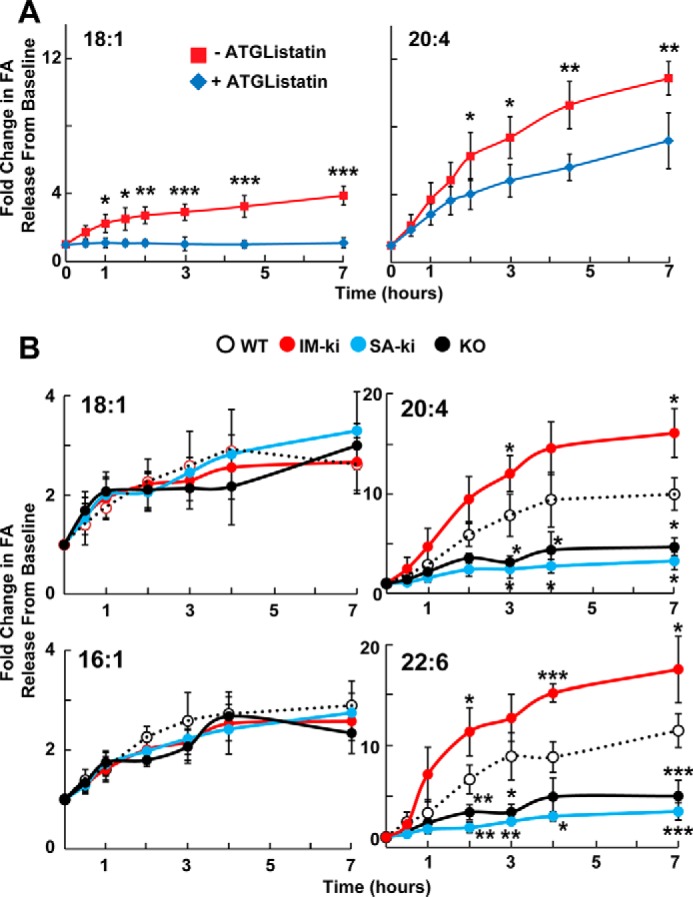
**Release of FAs from hepatic LDs incubated *ex vivo*.** *A*, effect of ATGListatin on release of oleate (18:1) and arachidonate (20:4) from hepatic LDs. LDs were isolated from WT male mice (*n* = 6/group, 15 weeks old) fed a HSD for 4 weeks. After a 12-h overnight fast, mice were refed for 4 h and then killed. Livers were harvested, and hepatic LDs were isolated and diluted to a total volume of 5 ml. Aliquots (50 μl) of LDs were transferred to a glass 96-well plate containing 250 μl of isolated liver cytosol, ATP-Mg (1 mm), and 0.5% albumin with or without 1 μm ATGListatin solution and warmed to 37 °C. For each liver, LDs were added to 24 wells with or without ATGListatin. The plate was then lightly vortexed and incubated at 37°C. At the indicated times, LDs were disrupted by the addition of 300 μl of methanol and stored at 4°C until they were extracted and analyzed by direct infusion TOF-MS. Values were normalized to the amount of FA in the medium at the 0-h time point and expressed as -fold change over baseline. The experiment was repeated with similar results. *B*, release of FAs by hepatic LDs from WT (*white*), IM-ki (*red*), SA-ki (*blue*), and PNPLA3 KO (*black*) male mice (*n* = 6/group, 15 weeks old). Mice were fed a HSD for 4 weeks, fasted overnight, and killed after 4 h of refeeding. Hepatic LDs were isolated and incubated *ex vivo* as described in *A* above, except that no ATGListatin was added. Values were normalized to the amount of FA in the medium at the 0-h time point and expressed as -fold change over baseline. The experiment was repeated once, and the results were similar. Each point represents the average of three mice, each of which was assayed in triplicate. *Error bars*, S.D. *, *p* < 0.05; **, *p* < 0.01; ***, *p* < 0.001.

Adipose TG lipase (ATGL) is the primary TG hydrolase in hepatocytes (30). To assess the contribution of ATGL to FA release from hepatic LDs, we monitored the release of FAs from hepatic LDs incubated *ex vivo* in the absence or presence of an ATGL inhibitor, ATGListatin (31). At each time point, the total amount of FA in the medium was expressed relative to the amount in the sample (medium and LDs) at time 0 (Fig. 9*A*). In the absence of ATGListatin, the amount of free MUFA (18:1) in the medium increased ∼4-fold (*left*), whereas free vLCPUFA (20:4) increased ∼10-fold (*right*). ATGListatin almost completely abolished the increase in free MUFA (18:1) (*left*). The release of vLCPUFAs (20:4) from LD was decreased ∼60% (*right*). Therefore, ATGL was responsible for almost all of the oleate that was released, whereas only ∼40% of the vLCPUFA (20:4) released from the LDs was ATGL-dependent.

If the release of MUFAs from hepatic LDs is inhibited by ATGListatin, then inactivation of PNPLA3 would be predicted to have little impact on the appearance of palmitoleate or oleate in the medium in this *ex vivo* LD hydrolysis assay. To test this hypothesis, we monitored release of the two FAs from LDs of WT, IM-ki, SA-ki, and KO mice. The rates of appearance of 16:1 or 18:1 in the medium did not differ among the four strains (Fig. 9*B*, *left*). This finding is consistent with the hypothesis that PNPLA3 plays little role in the hydrolysis of abundant TG-FAs such as oleate (18:1) and palmitoleate (16:1) in LDs. In contrast, significant differences were seen in the appearance of arachidonic acid (20:4) and docosahexenoic acid (22:6) into the medium. The amount of arachidonate in the medium of LDs from WT and IM-ki mice increased ∼10- and ∼15-fold, respectively. In contrast, arachidonate release from the LD of KO and SA-ki mice was significantly lower (3–5-fold). These data support the hypothesis that PNPLA3 functions as a vLCPUFA-specific TG hydrolase that promotes transfer of vLCPUFA from TG to PC and possibly other PLs on LDs.

## Discussion

The major finding of this study was that PNPLA3 promotes transfer of vLCPUFAs from TG to PLs in hepatic LDs and that this activity is enhanced in mice expressing the PNPLA3(148M) variant. The redistribution of vLCPUFA from TG to PC in WT mice depended on the integrity of the catalytic dyad of PNPLA3. In mice in which PNPLA3 had been inactivated, either by deletion of exon 1 (*Pnpla3*^−/−^ mice) or by substituting alanine for the catalytic serine (47A-ki mice), the converse pattern was seen: vLCPUFAs were enriched in TG and depleted in PLs (Fig. 2). Consistent with the premise that PNPLA3 catalyzes the transfer of vLCPUFA from TG to PL in LDs, overexpression of PNPLA3(WT) in HuH7 cells decreased incorporation of vLCPUFA into TG, increased vLCPUFA incorporation into PC, and had no effect on the incorporation of palmitate into either lipid (Fig. 8). Moreover, release of vLCPUFA from LD was reduced in LDs isolated from *Pnpla3*^−/−^ mice and 47A-ki mice, whereas it was increased in LDs from IM-ki mice when compared with WT animals (Fig. 9). Taken together, these data support the hypothesis that PNPLA3 is a lipase that acts on the surface of LDs, where it cleaves vLCPUFAs from TGs and makes them available for incorporation into PLs. In this model, the physiological role for PNPLA3 is to mediate the remodeling of TGs and PLs on LDs.

Previously Ruhanen *et al.* (24) found that vLCPUFA were enriched in PL from cultured hepatocytes (HuH7) expressing recombinant PNPLA3(WT) or PNPLA3(148M). Subsequent studies from the same group (32) and from Peter *et al.* (33) found that PNPLA3(148M) was associated with lower levels of arachidonate in hepatic TG. Those authors proposed that PNPLA3 plays a role in remodeling hepatic TG, which is consistent with the conclusions of the present study. They further proposed that PNPLA3(148M) causes hepatic steatosis due to a loss in this activity. The results of our study do not support this notion. The IM-ki and SA-ki mice have opposite effects on vLCPUFA distribution in LD, yet both accumulate liver TG on a HSD (22). Thus, the hepatic steatosis associated with PNPLA3(148M) does not appear to reflect a loss in the lipid remodeling activity of the enzyme.

The distribution and metabolism of vLCPUFAs in 148M-ki mice were consistent with an increase rather than a decrease in PNPLA3 activity. This finding appears to be in conflict with the marked reduction in TG hydrolase activity of purified recombinant PNPLA3(148M) (18). In that study, the *V*_max_ of PNPLA3(148M) was estimated to be ∼20% of the WT enzyme for TG, compared with <5% of WT for PNPLA3(47A) (18). Therefore, we expected that the lipid patterns in the LDs from the 148M-ki and 47A-ki mice would be similar, especially because both groups of mice accumulate PNPLA3 on LD and develop fatty liver (22, 23). Thus, there is a dissociation between PNPLA3 genotype and the partitioning of FA between TG and PLs in LDs, the accumulation of PNPLA3 on LD, and the liver fat content.

The reduced enzymatic activity of the PNPLA3(148M) may be offset by the massive accumulation of the mutant protein on LDs of IM-ki mice (22). The I148M variant causes an 80% reduction in PNPLA3 activity (18) but causes an ∼40-fold increase in the concentration of the enzyme on LD (22). The net result of these opposing changes may explain the increase in PL remodeling activity and enrichment of vLCPUFAs in the PLs of LDs in these animals. Alternatively, PNPLA3(148M) may have a higher affinity for the vLCPUFAs, resulting in enhanced FA transfer. The 47A-ki mutation of PNPLA3 also accumulates on LDs (22), but in contrast to the I148M substitution, replacement of the catalytic serine completely abolishes PNPLA3 TG hydrolase activity (18). Therefore, the accumulation of PNPLA3 in the SA-ki mice is not associated with an increase in PNPLA3-remodeling activity, so the distribution of vLCPUFA in hepatic LDs of the 47A-ki mice is similar to that of the KO animals.

Genetic ablation or inactivation of PNPLA3 decreased the vLCPUFA content of PL in LDs but did not decrease the total PL content of this organelle (Fig. 6). This finding is compatible with the notion that the PLs in LDs, like PLs in membranes (34), are initially synthesized primarily with saturated and MUFAs and are then remodeled on the droplet by incorporating vLCPUFA at the *sn*-2 position. The redistribution of vLCPUFA from TG to PLs was increased in mice expressing PNPLA3 (WT) (Fig. 6), indicating that PNPLA3 activity is a determinant of PL remodeling in LDs.

**Figure 10.**
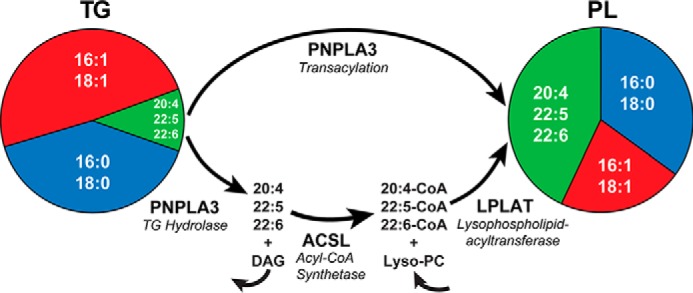
**Models of PNPLA3 function in FA remodeling.** PNPLA3 may act as a transacylase, transferring vLCPUFA directly from TG to lyso-PL. Alternatively, PNPLA3 may function as a vLCPUFA-specific TG hydrolase in a remodeling pathway for LD PL analogous to the Lands cycle for membrane PL. PNPLA3(148M) expression causes an increase in TG and PL remodeling, whereas PNPLA3(47A) has an opposite effect because it has no enzyme activity.

The biochemical mechanism by which PNPLA3 promotes transfer of vLCPUFA from TG to PLs in LDs is not known. Two potential pathways are shown in Fig. 10. First, PNPLA3 may transfer vLCPUFAs directly from TG to PL in LDs (transacylation). Jenkins *et al.* (16) and Huang *et al.* (18) found that PNPLA3 catalyzes transacylation of FAs among TG, diacylglycerides, and monoacylglycerides. CoA-independent transacylation of lyso-PLs has also been described (35), but the enzyme(s) involved in this reaction has not been identified. We are not aware of any evidence that PNPLA3 can promote CoA-independent transfer of FAs from glycerolipids to lyso-PL. It has been proposed that the CoA-independent transacylation system involves a phospholipase A2 (36), but PNPLA3 had little or no phospholipase activity in *in vitro* assays (16, 18). Further studies will be required to define the specific biochemical mechanism of PNPLA3-mediated transfer of vLCPUFA from TG to PLs.

An alternative role for PNPLA3 in the transfer of vLCPUFAs from TG to PL may be to catalyze the release of vLCPUFA from hepatic LDs (Fig. 10, *bottom pathway*). The released vLCPUFAs would be available to a lysophospholipid acyltranferase to incorporate into a PL. This model is consistent with *in vitro* studies of purified recombinant PNPLA3 (18) and with the established role of PNPLA2 (ATGL), the PNPLA family member most closely related to PNPLA3, as a TG hydrolase (37). Moreover, release of vLCPUFAs from LDs incubated *ex vivo* was reduced in LD from *Pnpla3*^−/−^ or SA-ki mice and increased when LDs from IM-ki mice were used in the experiment (Fig. 9*B*). These results suggest that PNPLA3 hydrolyzes vLCPUFA from TG.

In either of these models, completely removing PNPLA3 activity, by KO or 47A-ki, causes an increase in TG-vLCPUFA (Fig. 2) and a decrease in PL-vLCPUFA (Figs. 6 and 7). Conversely, increased PNPLA3 activity causes a decrease in TG-vLCPUFAs and increase in PL-vLCPUFAs. The I148M substitution causes an ∼15 reduction in protein activity but an ∼40-fold increase in LD PNPLA3. Together, this results in an ∼4-fold increase in total PNPLA3 activity in the 148M-ki mice, as demonstrated by the changes in lipid composition (Figs. 2, 6, and 7). These changes in composition are restricted to LDs and do not affect the whole-organ PL composition.

We cannot exclude the possibility that PNPLA3 has other biochemical activities that are important for vLCPUFA transfer. We showed previously that PNPLA3 has thioesterase activity *in vitro*, and this activity is largely preserved in the 148M protein and abolished in the S47A protein (18). Although the physiological significance of this thioesterase activity is not known, it supports the notion that PNPLA3 may act by mechanisms other than TG hydrolysis. It is possible that PNPLA3 acts as a cofactor or scaffolding for a protein associated with the sorting of FAs on LDs. Alternatively, it could affect the elongation or desaturation of FAs targeted to LD subclasses.

The remodeling of PLs in LDs by PNPLA3 appears to be distinct from the PL remodeling that occurs by the so-called Lands pathway. In that pathway, vLCPUFAs are transferred from PLs to the *sn*-2 position of lyso-PLs (29). Transfer of the acyl group requires both ATP and CoA, and it involves formation of an acyl-CoA intermediate. Kumari *et al.* (38) reported that PNPLA3 catalyzed the transfer of FAs from acyl-CoA to lysophosphatidic acid, but this activity did not require the catalytic serine and is therefore distinct from the PNPLA3 activity described in the present study (38).

The activity of PNPLA3 on the vLCPUFA content of hepatic lipids appears to be restricted to LDs, which contain virtually all of the TG but only a small fraction of the PLs in cells. PNPLA3 is not required for remodeling the major bulk of PL in cell membranes but may instead catalyze a novel PL remodeling pathway that takes advantage of the abundance of TG in LDs to provide a source of vLCPUFA. The finding that PNPLA3 is strongly up-regulated by feeding is consistent with this notion, because vLCPUFAs are diet-derived and are rapidly incorporated into liver lipids (39).

The physiological significance of incorporating vLCPUFA at the *sn*-2 position of PLs is not fully understood. One possibility is that vLCPUFA-containing PLs are required to accommodate the growth of LDs during feeding. Incorporation of vLCPUFAs into LD-PLs would be predicted to decrease the surface tension of the droplets and could thereby facilitate budding or stabilization of cytosolic LDs (40). Alternatively, incorporation of vLCPUFA into droplet PL may also influence the protein composition of the droplet surface (*e.g.* to appropriately balance rates of TG synthesis and hydrolysis in accordance with cellular energy needs). In this model, proper LD-PLs would be required for recruitment and activation of LD-stabilizing protein (*e.g.* perilipins). A third possibility is that vLCPUFAs are retrieved from TG and sequestered in PLs to limit the loss of essential FAs through β-oxidation. This process would be most relevant for diets deficient in essential FAs. PNPLA3's role in postprandial sorting of vLCPUFAs from TG to PLs is consistent with these models. Regardless of how PNPLA3 is involved in vLCPUFA sorting and lipid remodeling, this activity is not directly related to PNPLA3-induced nonalcoholic FLD because the 148M and 47A have opposite effects on this activity, but both cause diet-induced FLD.

## Experimental procedures

### Mice

I148M and S47A were introduced into mouse *Pnpla3* by homologous recombination as described by Smagris *et al.* (22). *Pnpla3*^−/−^ mice (KO) mice were obtained from Erin Kershaw (19). WT mice were generated from heterozygote breeding of either IM-ki or SA-ki mice. All mice were bred onto a C57BL/6J background (N6 for IM-ki; N5 for SA-ki). Mice were maintained on a 12-h light/dark cycle and fed Teklad Mouse diet 7001 (Harlan Teklad) after weaning. Male mice were used for all experiments. All animal experiments were performed with the approval of the Animal Research Advisory Committee at the University of Texas Southwestern Medical Center (Dallas, TX).

Four weeks before each experiment, mice were switched to a fat-free HSD (catalog no. 901683; 74% kcal from sucrose, MP Biomedicals). For the 3 days immediately preceding each experiment, mice were metabolically synchronized by withdrawing food from 7 p.m. to 7 a.m. and providing food from 7 a.m. to 7 p.m. On the day of the experiment, mice were fed a HSD for 4 h before sacrifice.

### LD isolation

Lipid droplets were isolated using the method of Smagris *et al.* (22). Briefly, livers were resected from isofluorane-anesthetized mice, washed in ice-cold PBS, and homogenized in a glass Dounce homogenizer (Sigma) in 10 ml of buffer A (250 mm sucrose, 20 mm Tricine, pH 7.8) with protease inhibitors (Complete Mini EDTA-free protease inhibitor mixture, Roche Applied Science). Homogenized samples were centrifuged at 100 × *g* for 30 min to remove debris. The supernatant (∼7 ml) was layered under 4 ml of buffer B (100 mm KCl, 2 mm MgCl_2_, 20 mm HEPES, pH 7.4) plus protease inhibitors. Samples were then centrifuged in a swinging bucket rotor (TH-641, Sorvall) at 17,700 × *g* for 30 min at 4 °C. After ultracentrifugation, the lipid layer was removed. The remaining material was subjected to centrifugation at 13,000 × *g* for 5 min before removal of the bottom phase (1 ml); this process was repeated three times. The top 1 ml of the final spin was removed and used for further analysis.

### Total hepatic TG analysis

Lipids were extracted from ∼50 mg of liver using a Folch extraction (41). Tissue levels of TG were measured using an enzymatic assay (Infinity, Thermo Electron Corp. and Wako Inc.) and normalized to sample weight.

### Lipidomics

Lipids were extracted using a modified Bligh–Dyer extraction for most of the analyses performed (42). Briefly, samples were homogenized in methanol (MeOH) using a Bead Beater (Omni International) and then diluted to a volume of 10 ml. An aliquot corresponding to 100 μg of tissue was removed and added to 8 ml of 1:1:2 PBS/MeOH/dichloromethane (DCM). The mixture was vortexed and centrifuged, and the bottom layer was removed. A total of 4 ml of DCM was added to the top layer, vortexed, and centrifuged, and the bottom layer was pooled with the previous bottom layer before the material was dried under a light stream of N_2_ and resolubilized in solvent. For untargeted lipidomics, samples were resolubilized in 1:1:2 isopropyl alcohol (IPA)/MeOH/DCM, 5 mm ammonium fluoride. For targeted TG and RE analyses, the samples were solubilized in MeOH. For targeted PL analysis, samples were solubilized in hexanes.

The LD-PLs were separated from neutral lipids using a three-phase extraction (28). LDs were added to a 4:4:4:3 solution of PBS/methyl acetate/hexanes/acetonitrile (10 ml). Samples were vortexed and centrifuged, and the top and middle layers were removed. The top layer, composed primarily of hexanes, contained primarily neutral lipids. The middle layer was enriched with PLs.

### Untargeted shotgun analysis

Lipid extracts were directly infused over a 4-min interval into a Sciex 5500+ triple TOF MS at a rate of 10 μl/min. The infusion began with a TOF product ion scan of 30 s. After the initial TOF collection, precursor ions were collected in an MS/MS^ALL^ style sequential product ion analysis where the fragmentation pattern of each unit mass window between 150 and 1200 Da was collected. This process was repeated in positive and negative mode. After acquisition, lipids were identified based on their tandem mass spectral features. The intensity value of each lipid was then normalized to the total ion signal for the entire run or to an internal standard. For some analyses, the sum of lipids with common features (*e.g.* number of double bonds) was used.

### GC-flame ionization detection analysis of FA composition

Total FA composition was analyzed by GC-flame ionization detection (27). Briefly, lipids from ∼50 mg of liver were extracted by a modified Folch extraction. The lipids were then saponified, reextracted, and derivatized with trimethylsilane. FAs were separated and detected using a Hewlett Packard 6890 series gas chromatograph. Fatty acids were identified based on the retention times from a purified FA standard mixture (GLC-744 Nu-Chek Prep) and normalized to an internal standard of pentadecanoic acid (C15:0).

### Targeted lipid analysis

TGs were measured using a Shimadzu LC20 HPLC coupled to a Sciex 5000 triple quadrapole MS. TGs were separated on an Agilent Poroshell 120 EC-C18 column (2.1 × 150 mm, 2.7-μm particles) and eluted with a solvent gradient transitioning from 98% MeOH, 2% DCM to 88% MeOH, 12% DCM over 22 min at a flow rate of 0.5 ml/min. The TGs were ionized by electrospray at an ion spray voltage of 3,500 V and temperature of 350 °C. The TGs were analyzed using multiple-reaction monitoring (MRM) mass pairs based on the MS1/MS2 combinations of the ammoniated adduct. The MRM pairs shown were 876.7/577.6, 848.7/549.6, 874.7/575.6, 898.7/577.6, 924.7/577.6, and 922.7/577.6 Da for TG 52:2, 50:2, 52:3. 54:5, 56:6, and 56:7, respectively. Each sample contained ∼50 μg of mouse liver homogenate with 1 ng of glycerol-triheptadecanoate (TG: C51:0) added as an internal standard (NuChek Prep). Values were normalized to the sum of all TG signals.

### Analysis of REs

REs were measured in MeOH/DCM extracts of mouse liver by the LC-MS/MS system described above. REs were separated on an Agilent Poroshell 120 EC-C18 column (2.1 × 150 mm, 2.7-μm particles) with a solvent gradient transitioning from 100% MeOH to 85% MeOH, 15% DCM over 30 min at a flow rate of 0.5 ml/min. The analytes were ionized by electrospray at a voltage of 4,500 V and temperature of 125 °C. Higher temperatures caused in-source decay of the REs, which limited specificity. REs were analyzed based on MRM pairs and identified by their characteristic 269.2-Da MS2 fragment. The total RE levels in livers of mice fed the fat-free diet were ∼100-fold lower than those from mice fed standard chow. The most prevalent RE species, RE16:0, RE22:5, and RE22:6, had MS1s of 541.8, 615.8, and 613.8 Da, respectively. Less abundant REs (*n* = 11) were also measured in this analysis. Authentic standards for RE16:0, RE22:5, and RE22:6 (Sigma) were used for chromatographic identification and to construct a calibration curve and establish a relative response ratio for absolute quantitation. For all other REs, the intensity was quantified against RE17:0 and summed as total REs.

### Analysis of PLs

Targeted analysis of PC, PE, PS, PG, and BMPs was performed by normal-phase LC-MS/MS, adapted from Hutchins *et al.* (43), with a Sciex 5000 triple quadrapole MS in line with a Shimadzu LC20 HPLC. Briefly, lipids from the middle layer of a three-phase extraction (see above) were separated on a Supelco Ascentis SI column (15 cm × 2.1 mm, 5-μm particles). Elution was initiated using 42% hexanes (Hex), 56% IPA, and 2% water for 5 min, followed by linear transitioning to 40% Hex, 54% IPA, and 6% water over 10 min. This was followed by a 5-min linear gradient to 38% Hex, 52% IPA, 10% water, which was maintained for 20 min before refreshing the column. The flow rate was set at 0.2 ml/min and maintained over the entire gradient. All solvents contained 5 mm ammonium acetate. Each sample was run twice, once in positive mode and once in negative mode, with an ion spray voltage of ±4,500 V and temperature of 300 °C. Each PL was analyzed based on MS1/MS2 MRM pairs. Positive-mode MS2 ions characteristic for PCs (184.1 Da) were used to identify PC species. Neutral losses of 171.1 Da were used for BMPs and PGs. In negative mode, the FA in the MS2 scan was used for all PL classes. Retention times for each lipid class were determined using natural isolated lipid mixtures from Avanti Polar Lipids. The signal intensity of the targeted vLCPUFA-containing lipid was normalized to an abundant PL from the same class.

### FA lipid incorporation in HuH7 cells

HuH7 cells (from Dr. Michael Gale, 2002) were plated at a density of 100,000 cells/60-mm dish. After 1 day, cells were infected with adenoviruses encoding PNPLA3(WT) or PNPLA3(148M). An empty virus was used as a negative control (15). After 2 days, the medium was changed to contain 10% newborn calf lipoprotein-deficient serum (NCLPPS) and incubated for 18 h. The medium was then supplemented with palmitate-*d*_31_ and arachidonate-*d*_8_ (Sigma) conjugated to FA-free BSA (1 μm). Cells (five dishes per time point) were harvested at the indicated times and quenched with MeOH. SPLASH standard mixture (Avanti Polar Lipids) (20 μl) was added to each harvested sample, and the lipids were extracted and analyzed by untargeted lipidomics as described above. The total incorporation of FA into PCs was calculated from the sum of the intensities of all features with a fragment ion of 184 Da and a mass shift of 31 (palmitate) or 8 (arachidonate) normalized to the PL standard in the SPLASH mixture. TGs that incorporated labeled FAs were identified by the mass difference between the precursor ion (greater than 800 Da in the MS1 scan) and the fragment ion: 304.3 for palmitate-*d*_31_ and 329.4 for arachidonate-*d*_8_. These values were summed and normalized to the internal standard in the SPLASH mix to determine total FA incorporation.

### Ex vivo LDs analysis

LDs were prepared as described above. During the isolation, each well of a 96-well glass plate was filled with PBS (200 μl) containing ATP-Mg^2+^ (1 mm) and albumin (0.5%), pH 7.4, plus 50 μl of cytosol solution (27) prepared as described. Cytosol and ATP were added because they greatly increased the rate of FA release. Half of the wells included ATGListatin (Cayman) (1 μm), an inhibitor of ATGL (31). Isolated LDs were divided into two equal aliquots; one was used for lipidomics analysis, and the other was used for this FA release assay. The aliquot for assay of FA release was diluted to 5 ml in PBS. Plates were warmed to 37 °C, and then 50 μl of LD was added to each well. For each LD preparation, FA release was determined at eight time points, with three replicate wells per time point (24 wells/LD preparation). The plates were incubated at 37 °C, and the reactions were terminated by the addition of 300 μl of MeOH. Heptadecanoic acid (C17:0, NuChek Prep) was added to each sample at the completion of the incubation. Lipids were extracted by adding 600 μl of DCM, mixing, centrifuging, and transferring the lower layer to another 96-well glass plate using a Hamilton Starlet liquid-handling robot (Hamilton). The levels of palmitate, oleate, arachidonate, and docosahexanoic acid were measured using TOF-MS (Sciex 5600+). Each sample was infused for 30 s, and data from the last 10 s of infusion were used for analysis. Values corresponding to each FA were normalized to the internal standard (C17:0) and expressed relative to the amount of FA at the initial time point. The average -fold change of the triplicates from each mouse was used to calculate the mean ± S.D. FA release for each genotype. Areas under the curves of FA concentrations plotted against time were calculated using a first-order kinetic model with Matlab's (Mathworks) curve fitting tool.

### Statistical analysis

S.D. values were calculated using either Matlab or Excel (Microsoft). *p* values were determined from a two-tailed *t* test using either Excel or Matlab.

## Author contributions

M.A.M., H.H.H., and J.C.C. all helped design and interpret experiments. M.A.M. performed and analyzed experiments. M.A.M., H.H.H., and J.C.C. jointly wrote and edited the manuscript.

## Footnotes

3 The abbreviations used are:

PNPLA3: patatin-like domain–containing protein 3
ATGL: adipocyte TG lipase
BMP: bis(monoacylglycerol) phosphate
CE: cholesteryl ester
DCM: dichloromethane
FA: fatty acid
FLD: fatty liver disease
Hex: hexanes
HSD: high-sucrose diet
HTGC: hepatic triglyceride content
IPA: isopropyl alcohol
ki: knock-in
KO: knockout
LD: lipid droplet
MRM: multiple-reaction monitoring
MUFA: monounsaturated fatty acid
PC: phosphatidylcholine
PE: phosphatidylethanolamine
PG: phosphatidylglycerol
PL: phospholipid
PS: phosphatidylserine
RE: retinyl ester
TG: triglyceride
vLCPUFA: very long chain polyunsaturated fatty acid
Tricine: *N*-[2-hydroxy-1,1-bis(hydroxymethyl)ethyl]glycine.
